# A framework for the simulation of individual glycan coordinates to analyze spatial relationships within the glycocalyx

**DOI:** 10.3389/fcell.2024.1519831

**Published:** 2025-01-07

**Authors:** Sarah Fritsche, Leonhard Möckl

**Affiliations:** ^1^ Department of Physics, Faculty of Sciences, FAU Erlangen-Nuremberg, Erlangen, Germany; ^2^ Max Plank Institute for the Science of Light, Erlangen, Germany; ^3^ Department of Medicine/CITABLE, FAU Erlangen-Nuremberg, Erlangen, Germany; ^4^ Deutsches Zentrum Immuntherapie, Erlangen, Germany

**Keywords:** glycocalyx, super-resolution microscopy, simulation, computational methods, sialic acids, ocular glycocalyx, mucins, quantitative biology

## Abstract

The glycocalyx is a dense and dynamic layer of glycosylated species that covers every cell in the human body. It plays crucial roles in various cellular processes in health and disease, such as cancer immune evasion, cancer immune therapy, blastocyst implantation, and functional attenuation of membrane protein diffusion. In addition, alterations in glycocalyx structure may play an important role in ocular surface diseases, e.g., dry eye disease. Despite the emerging importance of the glycocalyx, various aspects of its functional organization remain elusive to date. A central reason for this elusiveness is the nanoscale dimension of the glycocalyx in conjunction with its high structural complexity, which is not accessible to observation with conventional light microscopy. Recent advances in super-resolution microscopy have enabled resolutions down to the single-digit nanometer range. In order to fully leverage the potential of these novel methods, computational frameworks that allow for contextualization of the resulting experimental data are required. Here, we present a simulation-based approach to analyze spatial relationships of glycan components on the cell membrane based on known geometrical parameters. We focus on sialic acids in this work, but the technique can be adapted to any glycan component of interest. By integrating data from mass spectrometry and quantitative biological studies, these simulations aim to model possible experimental outcomes, which can then be used for further analysis, such as spatial point statistics. Importantly, we include various experimental considerations, such as labeling and detection efficiency. This approach may contribute to establishing a new standard of connection between geometrical and molecular-resolution data in service of advancing our understanding of the functional role of the glycocalyx in biology as well as its clinical potential.

## 1 Introduction

**FIGURE 1:**
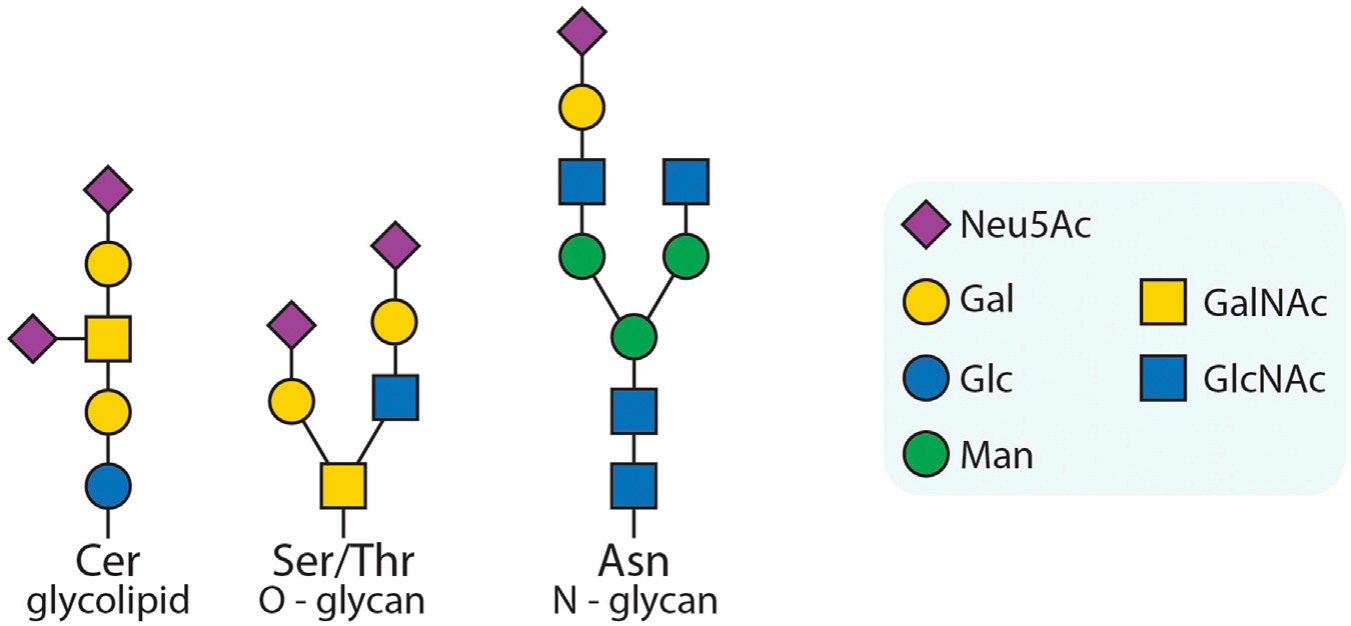
Exemplary glycans. Three examples of glycoconjugates: a glycolipid; an O-glycan; an N-glycan. The symbols are used according to the symbol nomenclature for glycans (SNFG) (Neelamegham et al., 2019). In this work, we will focus on sialic acids, a capping modification found on many types of glycans, as also depicted in the exemplary glycans here (purple squares). However, our approach can be adapted to other species as desired.

The glycocalyx is a dynamic, complex and dense layer of sugar structures that covers every human cell (Varki and Kornfeld, 2022). It consists of glycosylated species (Figure 1) that are linked to proteins, lipids, or RNA, thereby forming glycoproteins/proteoglycans, glycolipids, or glycoRNA, respectively. Additionally, polymeric glycan species such as hyaluronic acids are present, which are not covalently attached to another biomolecule. The glycocalyx is involved in numerous cellular and organismic processes, such as attenuation of membrane protein diffusion (Freeman et al., 2018) and immune system regulation (Hudak et al., 2014). Alterations in its structure can potentially disturb these vital processes, resulting in or contributing to diseases (Dennis et al., 2009; Reily et al., 2019). Therefore, both fundamental research and clinical applications will benefit from a deeper understanding of functional glycocalyx biology.

Despite previous insights, the glycocalyx remains, in comparison to the other two major classes of biomolecules, DNA and proteins, relatively underexplored. This is due to several challenges, which have impeded comprehensive investigations into functional glycocalyx biology. These include its sub-diffraction limit size, potentially disruptive sample preparation for electron microscopy (Chevalier et al., 2017), and the limited usability of classical genetic methods as glycocalyx components are secondary gene products. However, innovative strategies to image biomolecules (Reinhardt et al., 2023; Unterauer et al., 2024; Schueder et al., 2024) provide a new and exciting avenue for studying functional glycocalyx organization at previously inaccessible resolutions and with significantly expanded cellular context. As new and improved labeling approaches are expected, further increase in performance is anticipated. However, in order to fully leverage such novel experimental data, it is important to bridge the gap between previously known insights and the newly gained knowledge of cell surface organization. Furthermore, additional qualification and quantification of the robustness of the experimental results is required, as, for example, labeling efficiency strongly depends on the imaged cell type and might influence data interpretation (Luchansky et al., 2004).

There are several simulation projects that contribute to this task. GlycoSHIELD (Tsai et al., 2024) simulates glycan conformers onto static protein structures in order to predict the span of glycan shields. CHARMM-GUI (Jo et al., 2008) can generate representative, correctly oriented glycan structures using the GFDB database. With the help of a neural network, CandyCrunch (Urban et al., 2024) predicts glycan structures from tandem mass spectrometry data. The glycosylator (Lemmin and Soto, 2019) allows to create and analyze models of glycosylated proteins. DoGlycans (Danne et al., 2017) prepares glycans for analysis via atomic simulations. In addition, there are projects such as (Eduardo et al., 2014; Pikoula et al., 2018) which help to unravel the dynamics of the glycocalyx. While all of these projects are pushing the boundaries of our knowledge of the glycocalyx, a simulation capable of predicting potential distributions of specific structural units of glycans within the glycocalyx does not yet exist.

Here, we aim to provide the field with such a resource by developing a simulation pipeline as a starting point for future analysis of molecular resolution data in the context of the glycocalyx. We attempt to relate known structural features of the glycocalyx to topological arrangements as they might be extracted with advanced imaging methods, providing a framework for contextualizing experimental data. Due to the abundance of sialic acid residues in mammalian glycans and their considerable relevance in both fundamental and clinically applied glycosciences, we demonstrate our approach using this sugar (Smith and Bertozzi, 2021; Wisnovsky et al., 2021; Varki, 2008; Varki and Gagneux, 2012). However, we would to highlight that any sugar or glycan of interest can be investigated with our method.

## 2 Methods

In order to compare molecular resolution measurements with previously known data and to further quantify the obtained experimental results, the code provided ongithub was established. Here, we will give a general overview of the cornerstones of the procedure, which will help a user to get quickly started.

**FIGURE 2:**
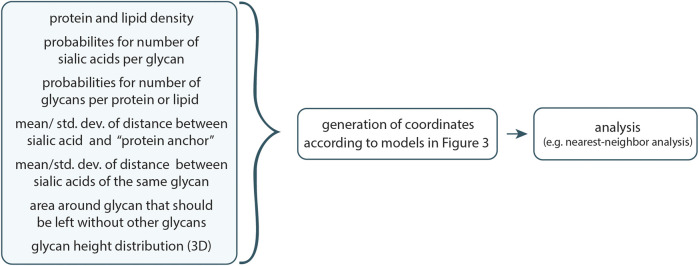
Workflow of the program. With the help of geometrical input, which can be easily tailored for each unique use-case, a possible set of coordinates of sialic acids is generated according to the models depicted in Figure 3. These coordinates can then be examined further, such as through a nearest-neighbor analysis.

The general workflow is visualized in Figure 2. Due to the stochastic nature of protein and lipid placement, as well as the precise distances between sialic acids and proteins, it is recommended to run multiple rounds of simulations to ensure consistency.

In the following, we delve more deeply into the specifics of the code starting with the determination of the number of glycoproteins and glycolipids within the field of view (FOV). The x- and y-coordinates of the glycolipid/glycoprotein centers are randomly generated, while the z-coordinate – in three-dimensional simulations – is determined by a skewed normal distribution.

**FIGURE 3:**
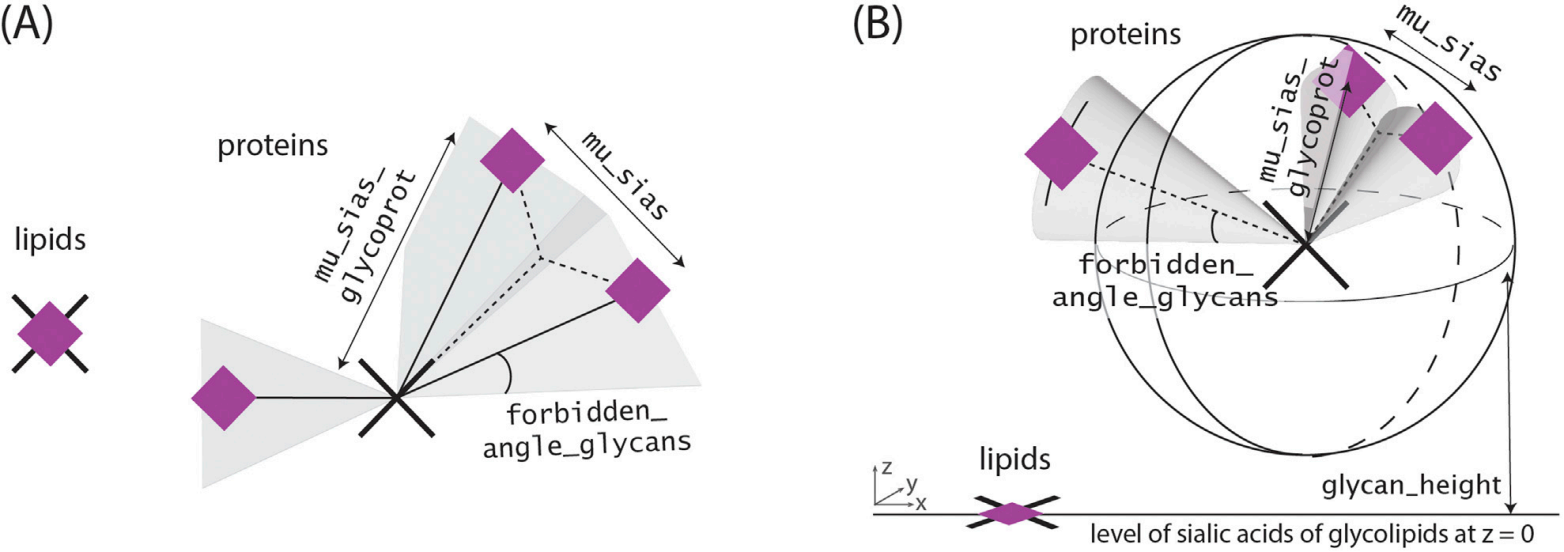
Glycoprotein and Glycolipid models. In both cases, the coordinates of the sialic acids from glycolipids are assumed to be identical with coordinates from the corresponding glycolipids, as each glycolipid typically has only a maximum of one glycan and one associated sialic acid. Sialic acids corresponding to the same glycan are located at an equal distance from the previously determined “protein anchor”, as the sugar-sequences before are oftentimes the same. We account for the glycans size as we do not allow for the placement of new glycans within a specific region around previously set sialic acids of one glycoprotein. **(A)** In the two-dimensional case this blocked region corresponds to the sections with an angle smaller than forbidden_angle_glycans besides the former set sialic acids. **(B)** In the three-dimensional case this blocked region corresponds to a cone by analogous definition. In the three dimensional case, all sialic acids are located in the upper sphere around the protein center as the cell membrane and protein occupy the bottom region.

Around these centers, the sialic acids are arranged according to Figure 3. Sialic acid positions from glycolipids and glycoproteins could be differentiated within the simulation, but as typical experimental data would not differentiate between them, their coordinate arrays are combined.

The code offers flexibility in selecting which steps to save during execution, thanks to the booleans boolean_save_ ∗ . For standard simulations, it is advisable to set boolean_save_coordstxt and boolean_save_analysistxt to True, as it allows future plotting and reconstruction of all relevant details. The flexible configuration of the code also provides visualization options and a nearest-neighbor analysis of the sialic acid coordinates. Each step of this process can also be reproduced individually.

The parameters of each simulation can be adapted to specific use cases through the “variables.py” file, where all length units are specified in microns. Specifically, these parameters are:

• global_name, path and variables_path: These string variables set the global name of the simulation, the saving directory and the path to the variable settings.
• FOV_x and FOV_y: These variables, which can be floats or integers greater than zero, define the area to be examined.
• prot_dens and lip_dens, representing the number of proteins and lipids per square micrometer, can be non-negative floats or integers.
• prob_x_glycoprots and prob_x_glycolips: These one-dimensional numpy arrays convey the probabilities of having no glycan on a given protein for the first entry, one glycan for the second, and so on. Their entries should sum up to one.
• prob_x_sias_prots and prob_x_sias_lips: Similar to the previous parameters, these one-dimensional numpy arrays specify the probabilities of having 0, 1, 2 …, sialic acids per glycan.
• mu_sias_glycoprot and sigma_sias_glycoprots refer to the distance between the sialic acid and its corresponding glycoprotein (see Figure 3). They determine the mean and standard deviation of the Gaussian distribution for the distance.
• forbidden_angle_glycans specifies, in radians, the angle around a glycan that must remain unoccupied, as shown in Figure 3.
• cutoff_gaussians is introduced in order to avoid unnaturally large distances. It represents the number of standard deviations considered to the left and right of the Gaussian means before cutting off the distribution.
• mu_sias and sigma_sias refer to the mean and the standard deviation of the distance distribution between two sialic acids as shown in Figure 3.
• mu_glycanheight, alpha_glycanheight, scale_glycanheight, lower_glycanheight and upper_glycanheight define the shape of the cut skew normal distribution, which determines the height of the “protein center” above the cell membrane (i.e., its z-coordinate). In case of mucin simulations, only lower_glycanheight and upper_glycanheight are important. The glycan height is then determined by a uniform distribution.

By changing these parameters, the simulation can be calibrated to best mimic specific experimental conditions, providing a bridge between hypothesis and observation.

To start a simulation, all input variables are manually adjusted in the “variables.py” file. The simulation can then be started in “main.py” using the routine() or the routine_3d() function. Their parameters can determine whether or not to save and plot the coordinates and (if applicable) their analysis, whether to analyze and if so for which labeling efficiencies and how often the array should be randomly reduced for each labeling efficiency. One can also change how many times the simulation should be repeated and, in addition, the default nearest neighbor distance from 1 to an arbitrary higher degree can be set. If several simulations are desired without the necessity of manual adjustment in between runs, the function change_variable() is useful, as it not only changes the content of the variables, but also writes the new values to the variable document, which is saved with each simulation round. At the beginning of each routine, the inputs are checked for logical errors to ensure that for example, the probability arrays indeed sum up to one.

While our code accurately simulates the basic structure of the glycocalyx, our code simplifies for efficiency. First, we have implemented a single Gaussian distribution for each distance, rather than combining several ones with different means, which would occur with different probabilities. Moreover, we consider an average over all glycan species within a simulation without sub-grouping according to, for example, number of glycans. However, this could easily be changed if necessary. Second, we do not yet consider non-capping (i.e., non-terminal) sialic acids, as they are a rather special case found in polysialic acid structures, but they could be added as well. Third, our model approximates proteins as spheres. This is, however, considered via the distance distribution between sialic acids and protein centers. Fourth, the code ensures that the first sialic acid of each glycan is within an “allowed region”, i.e., outside of the cones around previously set glycans. Therefore, there is the potential that, later, sialic acids are within a “forbidden region”. However, this only becomes a problem for a high number of sialic acids per glycan, rarely encountered in physiological cases.

While removing these simplifications is technically feasible, we opted against it to maintain a balance between model complexity, simulation run time, and the availability of necessary information. Nonetheless, the simulations continue to provide meaningful insights into the structure of the glycocalyx. As stated above, the code is easily adaptable to specific needs.

## 3 Results

**FIGURE 4:**
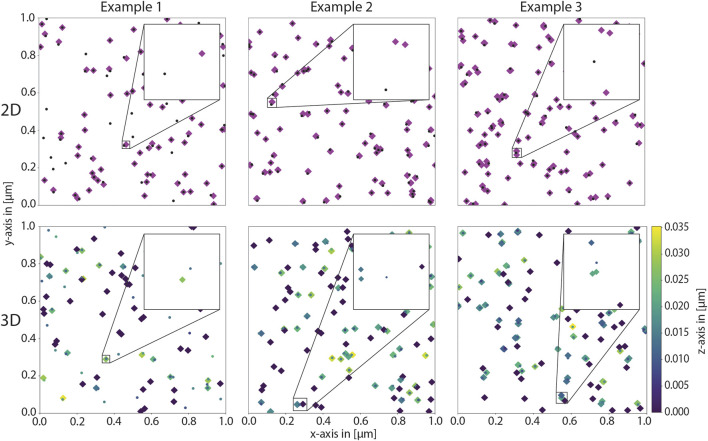
Benchmarking cases to asses model implementation. The protein density and the lipid density were set to 50 μm^-2^ in all examples. The top row shows the resulting coordinates of a two-dimensional simulation in the xy-plane, and the bottom row shows the resulting coordinates of a three-dimensional simulation, again in the xy-plane. The z-coordinate is color-coded according to height. In the left column, half of the glycans has one glycan with one sialic acid, the others do not have any glycans. In the middle, all proteins have exactly one glycan with two sialic acids each and on the right, half of the proteins has one and the other half two glycans where again, half of the glycans has one and the other half two sialic acids each. These distributions are observed in the results.

To test the robustness of the code, we simulated several benchmark cases, which are straightforward to assess visually. These include simulations in two and three dimensions. In the first example case, half of the proteins have exactly one glycan with exactly one sialic acid. In the second case, all proteins have exactly one glycan with exactly two sialic acids each. In the third case, half of the proteins has exactly one and the other half has exactly two glycans. Half of these glycans has exactly one and the other half exactly two sialic acids each. As evident from the coordinate distributions shown in Figure 4, the results match the expectation.

While visually interesting, scatter plots of point clouds often do not allow for a comprehensive understanding of the data, especially in complex cases. While there are many different ways to analyze such point clouds, a simple and straightforward way is to look at the nearest neighbor distance distribution. A nearest neighbor distance is the distance to the closest point next to the point of interest. Applied to all points, the resulting distribution of distances can, for example, give insights into the spatial homogeneity or clustering.

**FIGURE 5:**
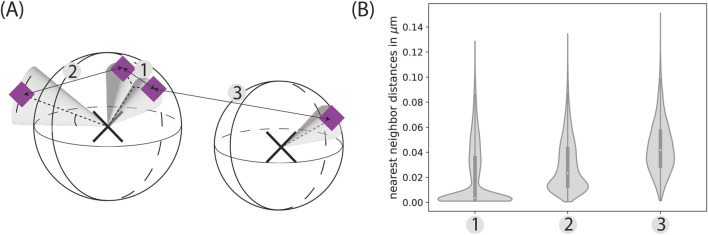
Characteristic distances. **(A)** Biologically, there are three characteristic distances: The distance between two sialic acids belonging to the same glycan (1), the distance between two sialic acids belonging to two different glycans located on the same protein (2), and the distance between two different glycoproteins (3). **(B)** These peaks can then be seen in the nearest neighbor distributions of coordinates simulated specifically to highlight these characteristic distances. The distributions are here depicted as violin plots with a corresponding box-plot in the middle, which indicates the median as well as the upper and lower quartile. The width of the distributions widens with increasing distance, as the distributions become less defined. While the peak of (1) corresponds to the distribution of *mu_sias* and *sigma_sias*, (3) varies greatly depending on the random distribution of the glycoproteins. The simulation parameters were set to a protein density of 125 µ $m^{-2}$ and a lipid density of 625 µ $m^{-2}$ within a 5 × 5 µm FOV. In (1) 70 % of the proteins have exactly 1 glycan, the rest none. Of these glycans, again 70 % have exactly two sialic acids, the rest none. In (2) 70% of proteins have two glycans and these glycans have a probability of 70 % for exactly one sialic acid. In the rest of the cases there are no glycans or sialic acids, respectively. In (3) 70% of the proteins have one glycan with one sialic acid in 70 %. The rest of the parameters is identical to Supplementary Table S2.

The specifics of the glycocalyx, in particular the distribution of sialic acids on glycans and proteins, lead to characteristic nearest neighbor distances of these point clouds, as highlighted by the simulations depicted in Figure 5. The peaks correspond to three distinct cases: (i) different sialic acids on the same glycan; (ii) sialic acids within different glycans on the same protein; and (iii) different sialic acids on distinct proteins.

**FIGURE 6:**
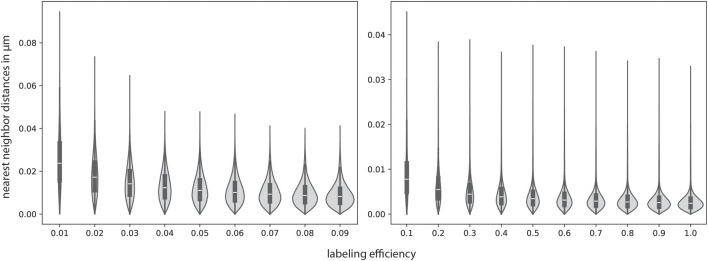
Sialic acid nearest neighbor distances simulated with biologically realistic parameters. The parameters were partly determined with GlycoSHIELD (Tsai et al., 2024) and vmd (Humphrey et al., 1996). All simulation parameters can be found in Supplementary Table S2. The nearest neighbor distributions are depicted as violin plots with a corresponding box-plot in the middle, which indicates the median as well as the upper and lower quartile. While the lowest nearest-neighbor distances stay approximately the same, the highest increase for lower labeling efficiencies, as expected.

Until now, we have only looked at theoretical benchmarking scenarios. In order to apply our approach to real-world biological cases, the parameter values that fit the respective case best have to be determined. To demonstrate a possible approach to this task, we choose values that are within biologically realistic parameters according to literature. We determined a possible lipid density by assuming the lipid heads to be spherical with a radius of 1 nm. It follows that a plane of lipids exhibits a lipid density of $2.9\cdot 10^{5}$ µ $m^{-2}$ . Since the protein to lipid ratio can be assumed to be between 1:50 and 1:100 (Cooper, 2000), we took a ratio of 1:75 to obtain a value for the protein density of $3.8\cdot 10^{3}$ µ $m^{-2}$ . It should be noted that these values are rather high estimates. Later, we will also discuss simulations with lower values. In order to determine the distance between two sialic acids, we used GlycoSHIELD (Tsai et al., 2024), a software package that can be used to model glycan-protein interactions using molecular dynamics simulations. We then measured the distances between the sialic acids of several glycans of interest within vmd (Humphrey et al., 1996). The measured distances were fitted with a Gaussian function, of which the mean and standard deviation was used for the simulation input. The distance between the sialic acids and the protein anchor varied between 1 and 3.4 nm (see Supplementary Table S1). Additionally, we have to account for the radius of proteins, which lies at minimum between 1 and 5 nm (Erickson, 2009). We thus chose a higher mean value of 12 nm. The other simulation values, all listed in Supplementary Table S2, were chosen to be in accordance to typical biological samples. As an analysis method, we chose to take a closer look at the nearest neighbor distributions depending on the labeling efficiency as depicted in Figure 6. The parameter of the labeling efficiency is critical in fluorescence microscopy as only imaging targets that carry a label can be visualized. Since the labeling efficiency is, due to experimental reasons, rarely 100 %, quantitative readouts of distance measurements must take reduced labeling efficiencies into account.

**FIGURE 7:**
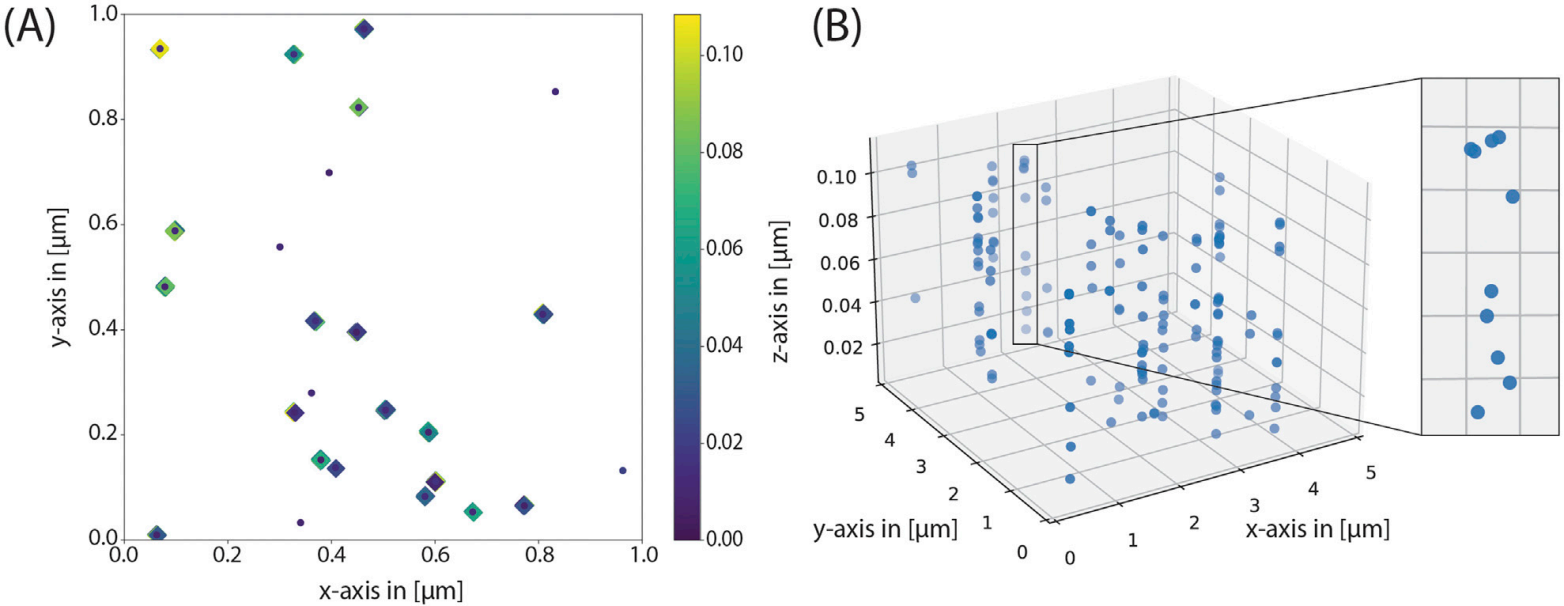
Mucin simulation. **(A)** In the distribution of coordinates can be seen that the mucins correspond to many sialic acids around the same glycoprotein in widely varying heights. **(B)** In a three-dimensional view, the tubular structure of the mucins is clearly visible.

To further demonstrate the versatility of our approach, which not only enables the attenuation of parameters, but also the straightforward adaptation of functions and/or variables, we turned to the simulation of mucins. Mucins are highly glycosylated, rod-like proteins, which can reach heights up to several 100 nm above the cell membrane. Therefore, we could not use the default model, where all glycans start at one “protein anchor”. Instead, we introduced a new function coordinates_grouped_around_3d_mucinextension(), which is almost identical to the previous one. However, the glycans are introduced at random heights within the lower and upper limit, specified in the variable file. For this, we had to merely introduce two auxiliary variables, and at merely two points in the code, the z-parameter had to be changed. Furthermore, we changed the number of glycans to higher values as they are encountered on mucins (up to 15 glycans per protein, which is rather low for mucins). Since we simulated mucins, the forbidden region was set to zero, as it follows from the biological architecture of mucins, that the glycans will be located at different heights and therefore their spatial orientation with regard to the protein backbone does not depend on the other glycans. With this approach, the stacked organization of sialic acids can be seen in Figure 7.

Finally, in order to showcase the adaptability of our method in the context of a specific area of glycocalyx research, we decided to study important representatives of the ocular glycocalyx.

For this example, we set the protein and lipid density to 500 and 2500 µ $m^{-2}$ , respectively. For the mucins present within the ocular glycocalyx as well, we chose three different lengths, which are present in equal parts, of 50, 250 and 500 nm. These numbers are within the range of typical mucins like MUC1, MUC4, MUC16 and MUC20. These mucins were previously shown to be on the stratified epithelia of cornea and conjunctiva (Mantelli and Argüeso, 2008; Woodward and Argüeso, 2014). Assuming a size of 0.36 nm for each amino acid (Dietz and Rief, 2006) and glycans on ≈ 20% of all amino acids, this corresponds to 28, 139 and 178 glycans on each of these mucins. These 20 % lie within the typical range of possible amino acids for O-glycosylation within the tandem repeat area of the mentioned mucins (Chaturvedi et al., 2008; Chen et al., 2021). Since glycans of the well-studied tear fluid are mainly O-linked glycans, we used these type of glycans for the distribution of sialic acids per glycan (Nguyen-Khuong et al., 2014). The O-glycan with two sialic acids that is present most frequently is DiSialyl-T (Nguyen-Khuong et al., 2014) with a distance of 2.5 nm, which we therefore took as the mean distance between two sialic acids. Since we simulated mucins, the forbidden region was set to zero and mu_, alpha_and scale_glycan height did not play a role in the simulations. The other parameters were set according to the previously mentioned reasonable parameters listed in Supplementary Table S2.

**FIGURE 8:**
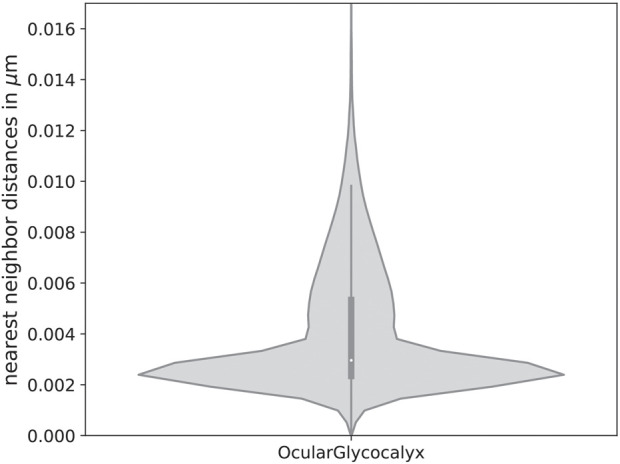
Nearest neighbor distribution of sialic acids on the ocular glycocalyx. The maximal values were cut in the diagram but lie at 0.046 µm and are due to the relatively sparsely set number of glycolipids with sialic acids, which are in rather high distance to other sialic acids. We simulated an equal distribution of three different mucin lengths of 50, 250 and 500 nm and 28, 139 and 178 glycans, respectively.

With these values, a nearest neighbor distribution like in Figure 8 arises. The distinct peak at low distances is a consequence of the mean distance between two sialic acids of the same glycan. The higher values arise due to a mixture of the distance between the distance of the protein center to the sialic acids and average height differences. Therefore, while the height of two consecutive sialic acids of one mucin can be rather similar, the horizontal position typically varies more, which explains the shoulder in the sialic acid distribution. Even higher values up to 46 nm, which were rarely observed and are not displayed in the figure, can be explained by the rather sparse number of glycolipids containing sialic acids.

## 4 Discussion

Overall, we have established a framework to simulate nanoscale structural features of the glycocalyx, namely, sialic acid distributions. While doing so, knowledge about its components from previous experiments is taken into account. By analyzing the localizations, these simulations can be compared to experimental results in order to verify them. In the future, we aim to expand and adapt the code further. For example, we aim to enhance the accessibility by providing a GUI. The interface will allow users to run simulations, adjust parameters and visualize results without needing to interact directly with the code, making it more accessible to researchers from all different backgrounds in this interdisciplinary research field. Furthermore, we would like to implement parallelization capabilities to allow computations of larger FOVs and more complex structures within a reasonable simulation time. As our framework evolves, it could be adapted to model additional components of the glycocalyx beyond sialic acids, according to the current experimental state.

In conclusion, our results serve as an essential step toward a comprehensive and quantitative model of the glycocalyx. By expanding the framework’s capabilities and fostering its accessibility to a broader range of researchers, we are optimistic that it will play a critical role in advancing our understanding of this complex nanoscale structure and we are looking forward to the research that is about to follow it.

## Data Availability

The code established within this study can be found in the github repository SimulationOfIndividualGlycanCoordinates.
