# Supplementary material for: A framework for the simulation of individual glycan coordinates to analyze spatial relationships within the glycocalyx
Source: Front Cell Dev Biol. 2025 Jan 7;12:1519831. doi: 10.3389/fcell.2024.1519831 (PMC11747212; doi:10.3389/fcell.2024.1519831)
Supplement: Supplementary file 1 [file DataSheet1.pdf]

Supplementary Material

1 SUPPLEMENTARY TABLES AND FIGURES

glycan	distances between sialic acids [nm]	distances to protein anchor [nm]
A2_26ANE5AC	1.572	0.989, 1.978
A2F2	3.383	2.208, 2.294
Gal2_Fuc2_Neu1	-	3.175
Fuc1_Neu4	2.137, 1.027, 3.029, 2.909, 3.567, 3.021	2.738, 2.599, 2.928, 2.037
Gal2_Fuc1_Neu1	-	2.368

Table S1. Typical distances within glycans. The table shows five typically encountered glycans and the distances between their sialic acids (if they have several) as well as the distance between the sialic acids and the protein anchor.

variable	value
`FOV_x`	1 µm
`FOV_y`	1 µm
`prot_dens`	$3.8 \cdot 10^3\,\mu m^{-2}$
`lip_dens`	$2.9 \cdot 10^5\,\mu m^{-2}$
`prob_x_glycoprots`	[0.3, 0.446, 0.164, 0.06, 0.022, 0.008]
`prob_x_sias_prots`	[0.3, 0.5, 0.2]
`prob_x_glycolips`	[0.6, 0.4]
`prob_x_sias_lips`	[0.65, 0.35]
`mu_sias_glycoprots`	0.012 µm
`sigma_sias_glycoprots`	0.002 µm
`mu_sias`	0.0026 µm
`sigma_sias`	0.0008 µm
`cutoff_gaussians`	2
`mu_glycan_height`	0.008 µm
`alpha_glycan_height`	2.5
`scale_glycan_height`	0.006 µm
`lower_glycan_height`	0.002 µm
`upper_glycan_height`	0.025 µm
`forbidden_angle_glycans`	10°

Table S2. Typical, biologically reasonable parameter setting, which can be used for the simulations.
